# Prolonged Cannabidiol Treatment Lacks on Detrimental Effects on Memory, Motor Performance and Anxiety in C57BL/6J Mice

**DOI:** 10.3389/fnbeh.2019.00094

**Published:** 2019-05-07

**Authors:** Eva M. Schleicher, Frederik W. Ott, Melanie Müller, Barbara Silcher, Marius E. Sichler, Maximilian J. Löw, Jannek M. Wagner, Yvonne Bouter

**Affiliations:** Department of Psychiatry and Psychotherapy, Division of Molecular Psychiatry, University Medical Center Goettingen (UMG), Georg-August-University, Goettingen, Germany

**Keywords:** cannabidiol, behavior, morris water maze, anxiety, cannabinoid system, cannabis

## Abstract

The Cannabis plant contains more than 100 currently known phytocannabinoids. Regarding the rising consumption of the non-psychotropic phytocannabinoid cannabidiol (CBD) in people’s everyday life (e.g., beauty products, food and beverages), the importance of studies on the influence of CBD on healthy humans and rodents is evident. Therefore, the behavioral profile of CBD was investigated with a battery of behavioral tests, including motor, anxiety, and memory tests after prolonged CBD treatment. Adult C57Bl/6J wildtype (WT) mice were daily intraperitoneally injected with 20 mg/kg CBD for 6 weeks starting at two different points of ages (3 months and 5 months) to compare the influence of prolonged CBD treatment with a washout period (former group) to the effects of long term CBD treatment (current group). Our results show that CBD treatment does not influence motor performance on an accelerating Rotarod test, while it also results in a lower locomotor activity in the open field (OF). No influence of CBD on spatial learning and long term memory in the Morris Water Maze (MWM) was observed. Memory in the Novel Object Recognition test (NORT) was unaffected by CBD treatment. Two different anxiety tests revealed that CBD does not affect anxiety behavior in the Dark-Light Box (DLB) and OF test. Although, anxiety is altered by current CBD treatment in the Elevated Plus Maze (EPM). Moreover, CBD-treated C57Bl/6J mice showed an unaltered acoustic startle response (ASR) compared to vehicle-treated mice. However, current CBD treatment impairs prepulse inhibition (PPI), a test to analyze sensorimotor gating. Furthermore, prolonged CBD treatment did not affect the hippocampal neuron number. Our results demonstrate that prolonged CBD treatment has no negative effect on the behavior of adult C57Bl/6J mice.

## Introduction

Over the last years, there has been growing interest in the therapeutic potential of the phytocannabinoid cannabidiol (CBD) occurring naturally in the plant *Cannabis sativa/indica*, commonly known as marijuana. Several studies showed that CBD is involved, among others, in immunomodulatory, anti-inflammatory, antiemetic, anticonvulsant, anxiolytic, antipsychotic, muscle relaxant and neuroprotective processes (Atakan, 2012; Burstein, 2015; Watt and Karl, 2017). Interestingly, the interest in studying CBD initially came through its interaction with the probably most commonly recognized constituent of the cannabis plant, 9-Tetrahydrocannabinol (THC). CBD was first isolated by Adams et al. (1940) in 1940 and its structure was elucidated 23 years later (Cunha et al., 1980). The pharmacological mechanisms of THC are the most well understood among the more than 100 other currently known phytocannabinoids (Mechoulam et al., 2014). Whereas THC is dependent from CB1-, and CB2-receptor binding, the mechanism of action for CBD is still not fully understood (De Petrocellis and Di Marzo, 2010). There are several other receptors that appear to be involved in the therapeutic effect of CBD, such as TRPV1-, PPARγ-, 5-HT1A-, and GPR55-Receptors (Zygmunt et al., 1999; Bouaboula et al., 2005; Russo et al., 2005; O’Sullivan, 2007; Ryberg et al., 2007).

Beneficial impacts of CDB on multiple diseases, such as multiple sclerosis (Mecha et al., 2013), brain ischemia (Schiavon et al., 2014; Mori et al., 2017) and epilepsy (Patra et al., 2019) have been shown in animal models.

Several studies support the beneficial effects of CBD for treating neuropsychiatric disorders, particularly affective disturbances as anxiety, depression and schizophrenia (Micale et al., 2013; Kucerova et al., 2014; Blessing et al., 2015). An anxiolytic effect has also been observed in healthy humans (Cunha et al., 1980). Recently, Stark et al. (2019) showed that early treatment with CBD can even prevent the appearance of schizophrenia-like deficits.

Surprisingly, few studies have examined the possible effects of prolonged CBD treatment on healthy mice. Studies of CBD effects have been mostly restricted to its acute effect and less is known about the efficiency after chronic CBD treatment. Therefore, the purpose of this study was to investigate the consequences of prolonged CBD treatment on the behavior of healthy C57BL/6J animals. In addition, we analyzed the effects of CBD on behavior after a washout period.

## Materials and Methods

### Animals and Drug Treatment

C57BL/6J mice (Jackson Laboratories, Bar Harbor, ME, USA) were used in this study with an equal distribution of male and female mice. All animals were handled according to the guidelines of the Federation of European Laboratory Animal Science Association (FELASA) and approved by the “Lower Saxony State Office for Consumer Protection and Food Safety” (LAVES). Mice were kept in individually ventilated cages (IVC, 32 × 16 × 14 cm; Tecniplast, Hohenpeißenberg, Germany) in groups up to five. Water and food were available *ad libitum*.

**Figure 1:**
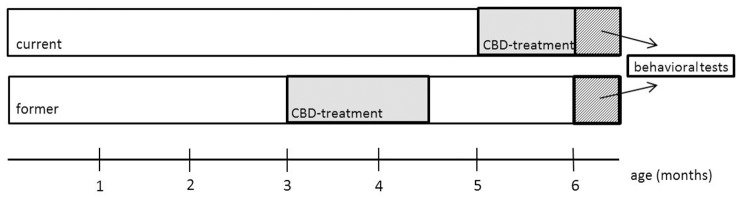
Schematic overview of the experimental design. At 3 (former) and 5 months (current), mice were treated daily with cannabidiol (CBD) for 6 weeks. The test battery started at 6 months for both groups. The current group was still treated with CBD during behavioral testing.

Powdered CBD (THC Pharm GmbH, Frankfurt/Main, Germany) was dissolved in equal amounts of 2.5 ml Tween 80 (Carl Roth GmbH, Karlsruhe, Germany) and 2.5 ml 100% ethanol and diluted in 45 ml of 0.9% NaCl solution. A vehicle control treatment group was set up in the exact same way, with the exception of CBD. Mice were assigned to either CBD or vehicle-treated groups and treated daily with an intraperitoneal injection containing the injection volume of 10 ml/kg body weight for 6 weeks starting at the age of 3 months (in the following called “former”) or 5 months (in the following called “current”; Figure 1). Mice were treated with 20 mg/kg body weight of CBD. Mice were weighed weekly and the injection volume was adjusted accordingly. In the current group, treatment continued during behavioral testing and lasted until the day of sacrifice. Behavioral testing started for all mice at the age of 6 months and mice were sacrificed with 26 weeks.

### Behavior Testing

To detect possible behavioral and cognitive alterations due to prolonged CBD treatment, C57BL/6J mice were tested in a battery of anxiety-, motor and memory-tests (*n* = 14–18). All mice were tested at the age of 6 months and testing lasted 18 days. Mice were sacrificed after the last day of testing.

Mice were kept on a 12 h/12 h inverted light cycle. All behavior experiments were performed during the dark phase between 7 a.m. and 7 p.m. Ambient illumination was 624 lx in the light chamber and 3 lx in the dark chamber. Red light illumination was 173 lx.

### Accelerating Rotarod

To analyze motor performance and motor skill learning in CBD-treated mice the accelerating Rotarod test (RotaRod 3375-5, TSE Systems GmbH, Bad Homburg, Germany) was used (Shiotsuki et al., 2010). C57BL/6J mice were placed face forwards on the rod (Æ 30 mm, 60 mm width per mouse), suspended above a grid floor at a height of 14.7 cm, high enough to create avoidance of falling and to prevent the mice deliberately jumping off the rod. Mice performed four trials per day on two consecutive days. To keep the mice focused on the task, the test was performed under red light. The rod accelerated from 4 to 40 revolutions per minute (rpm) over a time period of 300 s with an average inter-trial interval (ITI) of 15 min before the next trial started. For each trial, the time on the rod was recorded.

### Morris Water Maze

The Morris Water Maze test (MWM; Morris, 1984) was used to evaluate spatial reference memory in CBD-treated C57BL/6J mice as previously described (Bouter et al., 2014). In brief, the test relies on spatial cues to locate a submerged hidden platform (10 cm diameter) in a circular pool filled with non-transparent tap water. For spatial coordination, the pool was divided into four virtual quadrants that were defined based on their spatial relationship to the platform: left, right, opposite and target quadrant, which contains the goal platform.

During the cued training the platform was marked with a triangular flag and both, platform and starting position were changed in the four trials per day with an ITI of 15 min.

During acquisition training, spatial visual cues were fixed on the edge of the pool and the triangular flag was removed from the platform. This testing phase consisted of four trials per day over five consecutive days. Same trial procedures as for cued training were conducted.

The probe trial started 24 h after the last day of acquisition training to assess spatial reference memory at the end of the learning period. During the probe trial, the platform was removed from the pool and the mice were allowed to swim freely for 60 s.

To record escape latency, swimming speed and quadrant preference, ANY-Maze video tracking software (Stoelting Co., Wood Dale, IL, USA) was used.

### Open Field and Novel Object Recognition

The open field (OF) test was used to assess locomotor activity and exploratory behavior as described by Jawhar et al. (2012). During the OF test, mice were placed into a square box where they could freely explore the area for 5 min. ANY-Maze video tracking software was used to record the percentage of time spent in the central part vs. total time and total distance traveled during a single 5-min trial.

Twenty-four hours after later, Novel Object Recognition Test (NORT) was performed in the same box, now containing two identical objects during the first testing day. The NORT is a widely used test to assess memory and preference for novelty in rodents (Antunes and Biala, 2012). Mice were allowed to explore freely for 5 min. On day two of NORT, one of the objects was replaced by a novel object and the test time stayed the same. ANY-Maze video tracking software (Stoelting Co., Wood Dale, IL, USA) was used to record the distance traveled, percentage of time spent in the center and the exploration time of each object.

The percentage of exploration time for the novel object was calculated as follows:

$$Novel\;Object\;[\%]=\left(\frac{Novel\;Object}{Novel\;Object+Familiar\;Object}*100\right)$$

### Dark Light Box

The Dark Light Box (DLB) was used to test for possible anxiolytic- or anxiogenic-like effects of prolonged CBD in C57BL/6J mice. This test is based on the innate aversion of rodents to brightly illuminated areas and on the spontaneous exploratory behavior of rodents in response to mild stressors, such as a novel environment and light (Bourin and Hascoët, 2003). The test was performed using a gray plastic box (73 cm × 25 cm × 32 cm), which was divided into two areas: a smaller dark area (31 cm × 25 cm), covered by a black sheet made of plexiglass, and a larger light area (42 cm × 31 cm), which was not covered. These two areas were separated by a gray wall with a small opening (5 cm × 5 cm), allowing the mice to move freely from one compartment to the other. Each mouse was introduced into the light area facing the wall opposite of the small opening and was allowed to explore the space freely for 300 s. ANY-Maze video tracking software (Stoelting Co., Wood Dale, IL, USA) was used to record the time spent in each compartment and the number of line crossings.

### Elevated Plus Maze

The Elevated Plus Maze (EPM) test was used to assess anxiety-related behavior in C57BL/6J mice as previously described (Jawhar et al., 2012). The apparatus with a shape of a “+” consisted of four arms and a central area raised 75 cm above a padded surface.

Mice were placed in the center facing one of the two likewise oppositely positioned open arms and were allowed to freely explore the maze for 300 s. Distance traveled and the time spent in the open arms were recorded using ANY-Maze video tracking software (Stoelting Co., Wood Dale, IL, USA). Anxiety-like behavior can be measured by the time spent in the open arms as lower anxiety levels correspond to longer time spent in open arms (Karl et al., 2003).

### Prepulse Inhibition

The prepulse inhibition (PPI) was used as a test for sensorimotor gating (Pouzet et al., 1999). Each mouse was placed individually in a small metal grid cage (90 mm × 40 mm × 40 mm) to restrict exploratory behavior and major movements. The cage was equipped with a movable platform floor attached to a sensor recording vertical movements of the floor. The cage was placed in a sound-attenuating isolation cabinet (TSE GmbH, Bad Homburg, Germany). Each experimental session started with a 3 min habituation period to 65 dB background white noise (continuous throughout the session) followed by a 2-min baseline recording. Loudspeakers on both sides of the cage were used to induce startle reflexes by acoustic stimuli. A startle reaction to an acoustic stimulus including body muscle contractions and jumping causes movement of the platform. A transient force resulting from this movement was recorded during a time window of 100 ms beginning with the onset of the acoustic stimulus.

Six pulse-alone trials using startle stimuli of 120 dB and 40 ms were applied after the baseline recording. PPI was tested applying the 120 dB 40 ms startle pulse alone or preceded by a prepulse 20 ms stimulus of 70-, 75- or 80 dB. An interval of 100 ms with background noise was applied between each prepulse and pulse stimulus. Ten trials of startle response alone, no stimulus trials and pulse preceded by a 70-, 75- or 80 dB prepulse were applied in a pseudorandom order with ITIs from 8 to 22 s. Maximum amplitudes for all types of trials were averaged for every mouse. PPIs at each sound level were calculated using the following formula:

$$Prepulse\;inhibition\;[\%]=\left(1-\frac{average\;startle\;amplitude\;after\;prepulse\;and\;pulse}{average\;startle\;amplitude\;after\;pulse\;alone}*100\right)$$

### Quantification of Neuron Numbers Using Unbiased Stereology

Stereological analysis was used to obtain the overall neuron number in the CA1 region of hippocampus as previously described (Bouter et al., 2013). Briefly, the hemisphere was cut into coronal sections of 30 μm thickness, of which every tenth was systematically collected and stained with cresyl violet. The stereological analysis required a working station (Olympus BX51 with a motorized specimen stage for automatic sampling, StereoInvestigator 7; Microbrightfield, Williston, VT, USA). The CA1 region of sections from Bregma −1.34 mm to −3.80 mm were counted in, using a 100× oil lens (NA = 1.35). Neurons were counted with the optical dissector method; consequently, the total number of neurons was estimated by the optical fractionator method using a 2 μm top guard zone (West et al., 1991). The volume was calculated following Cavalieri’s principle (Rosen and Harry, 1990).

### Statistical Analysis

Differences between groups were tested with one-way and two-way analysis of variance (ANOVA) followed by Bonferroni multiple comparison or unpaired *t*-test as indicated. All data presented as mean ± standard error of the mean (SEM). Significance levels are set as follows: ****p < 0.0001; ***p < 0.001; **p < 0.01; *p < 0.05. All statistics were calculated using GraphPad Prism version 6.07 for Windows (GraphPad Software, San Diego, CA, USA).

## Results

### Cannabidiol Treatment Does Not Affect Motor Performance of C57BL/6J Mice

**Figure 2:**
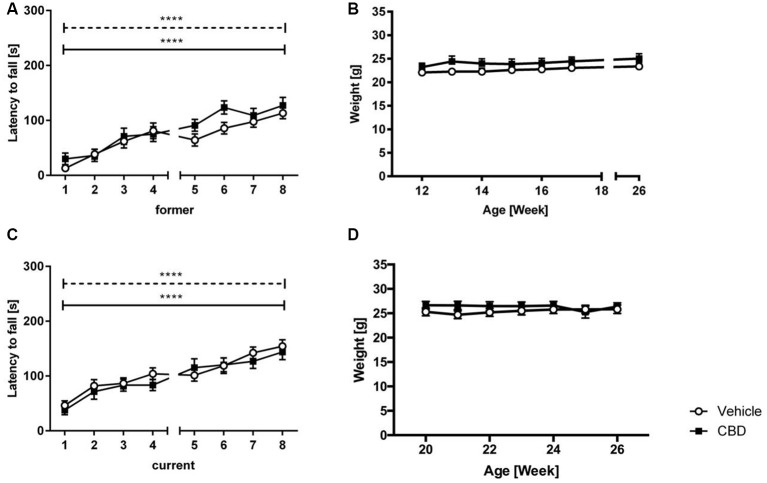
CBD treatment does not influence motor performance of C57BL/6J mice. Latency to fall **(A)** and weight **(B)** for former CBD-treated C57BL/6J mice. No significant treatment effect between vehicle and CBD in the current treated group **(C)**. Both groups showed an increased latency to fall over the training trials. No significant difference in weight in the current treatment group **(D)**. s = seconds, *n* = 14–19, ****p < 0.0001; two-way repeated measures analysis of variance (ANOVA) followed by Bonferroni multiple comparisons. Data presented as mean ± SEM.

No significant treatment effect was found in either former or current group (Figures 2A,C; two-way repeated measures ANOVA: *treatment* former: *F*_(1,27)_ = 1.792, *p* = 0.1919; *treatment* current: *F*_(1,36)_ = 0.7475, *p* = 0.393). Both vehicle and CBD-treated animals of the former and current treated groups showed a significant increase in motor performance over the eight trials (two-way repeated measures ANOVA: *trials* former: *F*_(7,189)_ = 23.02, *p* < 0.0001; *trials* current: *F*_(7,252)_ = 21.10, *p* < 0.0001).

### Cannabidiol Treatment Has No Influence on Body Weight

Former and current CBD-treated mice displayed similar weight compared to same-aged vehicle-treated mice over the 6 weeks of treatment (Figures 2B,D; two-way repeated measures ANOVA, *treatment* former: *F*_(1,27)_ = 1.045, *p* = 0.32; *treatment* current: *F*_(1,36)_ = 0.627, *p* = 0.434).

### Cannabidiol Treatment Does Not Alter Spatial Memory

**Figure 3:**
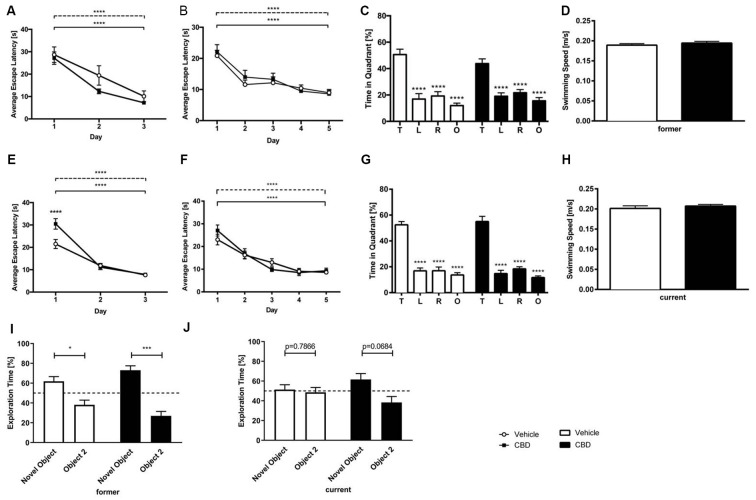
CBD treatment does not affect spatial learning and long term memory. CBD-treated and control mice of the former group displayed intact motor and visual performance in the Morris Water Maze (MWM; **A**). No impairment of spatial learning due to CBD treatment in acquisition training was seen in former treated mice **(B)**. The probe trial revealed that the long term memory was not affected by prolonged CBD treatment in C57BL/6J mice of the former group as they spent significantly more time in the target quadrant compared to the other quadrants of the maze **(C)**. The swimming speed during probe trial was not affected **(D)**. Current treated mice proved visual and motor abilities to swim in cued training **(E)**. Spatial learning was not altered as mice of the current group improved significantly during acquisition training **(F)**. In probe trial, current CBD-treated mice and vehicle-control mice displayed a clear preference for the target quadrant **(G)**. The swimming speed of the mice of the current group during probe trial did not differ significantly **(H)**. Furthermore, CBD treatment did not affect memory in novel object recognition (NOR) in the former group **(I)**. Whereas current CBD-treated mice **(J)** showed a trend towards novelty preference, former treated mice showed clear preferences for the novel object. Fifty percent chance level is indicated by a dashed line. Two-way **(A,B,E,F)** and one-way **(C,G)** ANOVA followed by Bonferroni multiple comparisons, unpaired *t*-test **(D,H)**, paired *t*-test **(I,J)**; *n* = 14–19, ****p < 0.0001; ***p < 0.001; *p < 0.05. Data presented as mean ± SEM.

In the cued training period, for both, former and current groups, vehicle and CBD-treated mice showed a significant decline in escape latency over time (Figures 3A,E; two-way repeated measures ANOVA, *days* former: *F*_(2,81)_ = 25.78, *p* < 0.0001; *days* current: *F*_(2,107)_ = 85.89, *p* < 0.0001). CBD-treated mice of the current group required significantly more time to find the platform only on day one than vehicle-treated mice (Figure 3E; two-way repeated measures ANOVA followed by Bonferroni multiple comparisons, *treatment day 1*: *p* < 0.0001). No overall differences in swimming speed could be detected between the age and treatment groups (data not shown; two-way repeated measures ANOVA, *treatment*). The cued training period revealed that all mice had an intact vision and the motoric abilities to swim.

Across the 5 days of acquisition training all animals, irrespective of treatment showed a significant decrease in the escape latencies (Figures 3B,F; two-way repeated measures ANOVA, *days* former: *F*_(4,134)_ = 17.49, *p* < 0.0001; *days* current: *F*_(4,174)_ = 29.65, *p* < 0.0001). During acquisition training no significant difference in swimming speed between the groups could be detected (data not shown; two-way repeated measures ANOVA, *treatment*).

### Cannabidiol Treatment Does Not Affect Spatial Reference Memory

Twenty-four hours after the last acquisition trial, a probe trial was performed to assess spatial reference memory. Both the vehicle- and CBD-treated mice of the former group displayed a significantly higher preference for the target quadrant, as indicated by the relative time spent in the different quadrants of the pool (Figure 3C; one-way repeated measures ANOVA followed by Bonferroni multiple comparisons, *former* vehicle: *F*_(3,52)_ = 59.81, *p* < 0.0001; Bonferroni for target quadrant vs. left, vs. right and vs. opposite quadrant: *p* < 0.0001; *former* CBD: *F*_(3,56)_ = 62.72, *p* < 0.0001, Bonferroni target quadrant vs. left, vs. right and vs. opposite quadrant: *p* < 0.0001). The swimming speed of the former group revealed no differences between the groups (Figure 3D; *former*: unpaired *t*-test, *F*_(14,13)_ = 1.29, *p* = 0.367).

In the same way, mice of the current CBD treatment group showed a significant preference for the target quadrant (Figure 3G; one-way repeated measures ANOVA followed by Bonferroni multiple comparisons *current* vehicle: *F*_(3,72)_ = 25.73, *p* < 0.0001, Bonferroni for target quadrant vs. left, vs. right and vs. opposite quadrant: *p* < 0.0001; *current* CBD: *F*_(3,72)_ = 20.88, *p* < 0.0001, Bonferroni target quadrant vs. left, vs. right and vs. opposite quadrant: *p* < 0.0001;). No difference in swimming speed was found among the current treatment groups (Figure 3H; *current*: unpaired *t*-test, *F*_(18,19)_ = 1.88, *p* = 0.368).

Our results show that prolonged CBD treatment does not impair spatial learning in C57BL/6J mice compared to vehicle-treated C57BL/6J mice, irrespective of the treatment time.

### Cannabidiol Does Not Affect Memory in the Novel Object Recognition Test

In the former treatment group, CBD-treated mice showed a clear preference for the novel object (Figure 3I; *former* CBD: paired *t*-test, *p* = 0.0002), which is seen in vehicle-treated mice, too (Figure 3I; *former* vehicle: paired *t*-test, *p* = 0.0263). In the current treatment group, CBD-treated mice showed a trend towards a preference for the novel object (Figure 3J; *current* CBD: paired *t*-test, *p* = 0.0684), whereas current vehicle-treated mice did not (Figure 3J; *current* vehicle: paired *t*-test, *p* = 0.7866).

### Cannabidiol Does Not Affect Anxiety Behavior in the Dark Light Box

**Figure 4:**
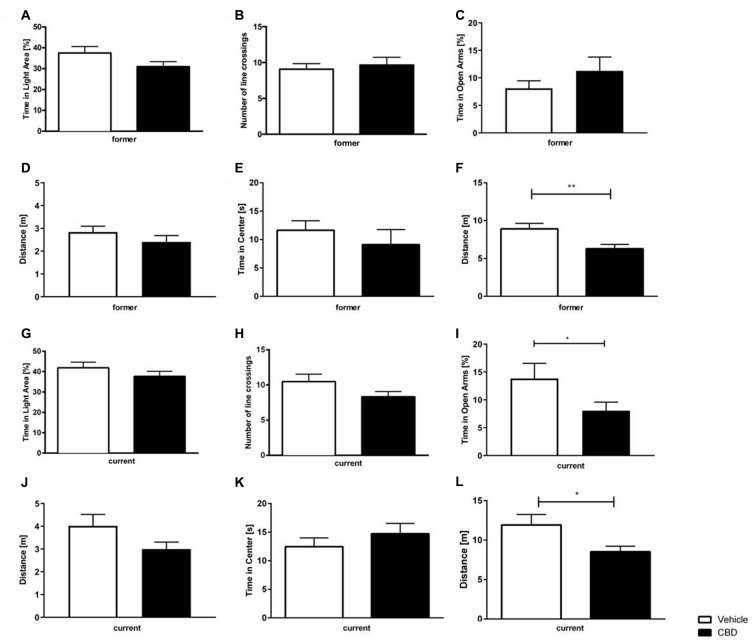
Effects of prolonged CBD treatment on anxiety-related behavior. No significant difference in time spent in the light area in former CBD-treated mice **(A)**. Number of line crossings as a confounding factor of mobility in the dark light box (DLB) did not significantly differ for the former treated group **(B)**. Time spent in the open arms expressed as a percentage of total time spent in the maze **(C)** and traveled distance in the elevated plus maze (EPM) for former treated mice **(D)**. In the open field (OF), there was no significant difference in time spent in the center of the box in former treatment group **(E)**. Thus, a higher locomotor activity for vehicle-treated mice was found in the former CBD-treated mice **(F)**. The time spent in the light area in current CBD-treated mice did not differ **(G)**, as well as the number of line crossings in the DLB **(H)**. Current CBD-treated mice showed a significant decrease in time spent in the open arms **(I)** but no difference in the distance traveled in the EPM **(J)**. No significant difference between current CBD-treated mice and their vehicle-treated littermates in the time in the center **(K)**, thus current CBD-treated mice traveled less distance in the OF **(L)**. Unpaired *t*-test; *n* = 14–19, **p < 0.01; *p < 0.05. Data presented as mean ± SEM.

In both, the former and current treatment group, CBD-treated mice did not explore the light box longer than the vehicle-treated mice (Figure 4A; *former*: unpaired *t*-tests,: *F*_(12,14)_ = 1.502, *p* = 0.101; Figure 4G; *current*: unpaired *t*-tests: *F*_(18,17)_ = 1.44, *p* = 0.464). Regarding the former group, CBD-treated mice crossed the line slightly more often than vehicle mice (Figure 4B; *former*: unpaired *t*-test, *F*_(13,13)_ = 2.03, *p* = 0.672). In the current treatment group, there was no difference measured in the number of line crossings observed between CBD and vehicle-treated mice (Figure 4H; *current*: unpaired *t*-test, *F*_(18,17)_ = 2.47, *p* = 0.095).

### Current Cannabidiol Treatment Alters Anxiety Behavior in the Elevated Plus Maze

Former CBD-treated animals did not show a significant difference in the amount of time spent in the open arms compared to vehicle-treated controls (Figure 4C; *former*: unpaired *t*-test, *F*_(14,13)_ = 3.759 *p* = 0.2856). In contrast, current CBD-treated mice showed a significant decrease in the time spent in the open arms vs. vehicle-treated control mice (Figure 4I; *current*: unpaired *t*-test, *F*_(18,19)_ = 3.344, *p* = 0.0134). The distance traveled was investigated to assess the confounding factor of movement. No significant difference in the distance traveled could be observed between the two different treatment groups, either with former or current treatment (Figure 4D; *former*: unpaired *t*-test *F*_(14,13)_ = 1.247, *p* = 0.2642; Figure 4J; *current*: unpaired *t*-test *F*_(18,19)_ = 2.850, *p* = 0.0969).

### Prolonged Cannabidiol Treatment Results in Lower Locomotor Activity in the Open Field

No significant difference between former vehicle and CBD-treated groups in regard to the time spent in the center could be detected (Figure 4E; *former*: unpaired *t-test*, *F*_(14,12)_ = 2.890, *p* = 0.4435). However, there was a significant difference in the distance traveled for a former treated vehicle vs. the CBD-treated group (Figure 4F; *former*: unpaired *t*-test, *F*_(13,14)_ = 1.441, *p* = 0.0081).

Similarly, current vehicle-treated mice traveled significantly more than CBD-treated mice (Figure 4L; *current*: unpaired *t*-test, *F*_(19,18)_ = 3.861, *p* = 0.0258), but did not spend significantly more time in the center than current vehicle-treated mice (Figure 4K; *current*: unpaired *t*-test, *F*_(19,18)_ = 1.235, *p* = 0.4084).

### Cannabidiol Treatment Does Not Alter Acoustic Startle Response in C57BL/6J Mice

**Figure 5:**
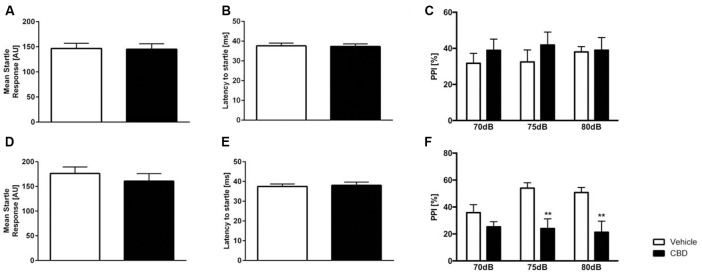
Altered prepulse inhibition (PPI) in CBD-treated C57BL/6J mice. Former **(A)** and current **(D)** CBD-treated C57BL/6J mice showed an unaltered acoustic startle response (ASR) and latency to startle **(B,E)**. PPI (PPI%) was unaltered in former CBD-treated C57BL/6J **(C)**. PPI was significantly altered in current treated CBD C57BL/6J mice at 75 dB and 80 dB **(F)**. Two-way ANOVA, *n* = 12–15, **p < 0.01. Data presented as mean ± SEM.

The acoustic startle response (ASR) to the startle stimulus alone was measured in CBD- and vehicle-treated C57BL/6J animals (Figure 5). Former (Figure 5A) and current (Figure 5D) CBD-treated C57BL/6J mice showed an unaltered startle response compared to same-aged vehicle C57BL/6J mice (*former*: unpaired *t*-test, *F*_(14,11)_ = 1.960, *p* = 0.2672; *current*: unpaired *t-test*, *F*_(14,14)_ = 1.754, *p* = 0.3048). Furthermore, the latency to startle was comparable between CBD and vehicle-treated animals (Figure 5B *former*: unpaired *t*-test, *F*_(14,11)_ = 2.124, *p* = 0.8995; Figure 5E *current*: unpaired *t*-test, *F*_(14,14)_ = 1.547, *p* = 0.801).

### Current Cannabidiol Treatment Impairs Prepulse Inhibition in C57BL/6J Mice

Current CBD wildtype (WT) treated mice displayed a significantly lower PPI compared to same-aged WT animals at 75 dB and 80 dB (Figure 5F; two-way repeated measures ANOVA: *treatment* current:* F*_(1,28)_ = 10.95, *p* = 0.0026; Bonferroni multiple comparison, vehicle vs. CBD: 75 dB *p* < 0.05 and 80 dB: *p* < 0.01). In contrast, former CBD treatment did not affect PPI (Figure 5C; two-way repeated measures ANOVA, *treatment* former: *F*_(1,25)_ = 0.5182, *p* = 0.4783).

### No Adverse Effect of CBD on Hippocampal Neuron Number

**Figure 6:**
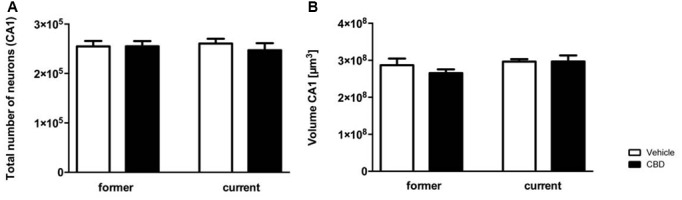
Prolonged CBD treatment does not affect hippocampal neuron numbers and volumes in C57BL/6J mice. The CA1 region was counted from Bregma −1.34 mm to −3.80 mm. Former as well as current CBD treatment had no influence on the total number of neurons in the CA1 region **(A)**. The CA1 volumes of the CBD and vehicle-treated mice in the former and the current group did not differ significantly **(B)**. One-way ANOVA **(A,B)**; *n* = 9–11. All data presented as mean ± SEM.

Design-based stereological analysis revealed that the hippocampal neuron numbers of the CA1 region did not significantly differ after former and current prolonged CBD treatment compared to the vehicle-treated mice (Figure 6A, one-way repeated measures ANOVA, *F*_(3,36)_ = 0.224, *p* = 0.879, former vehicle = 254,750 ± 11,038, former CBD = 255,240 ± 10,437; current vehicle = 260,663 ± 9,635, current CBD = 247,134 ± 14,129).

Likewise, no significant difference in volume of hippocampal CA1 region could be observed in the former or current group (Figure 6B; one-way repeated measures ANOVA, *F*_(3,36)_ = 1.198, *p* = 0.324; former: vehicle = 2.869e+008 ± 1.806e+007, CBD = 2.655e+008 ± 1.005e+007; current: vehicle = 2.965e+008 ± 6.610e+006, CBD = 2.967e+008 ± 1.634e+007).

The average number of sections counted was nine with a base of 196 μm and an optical dissector height of 5 μm. The average number of sections counted was nine with a base of 196 μm and an optical dissector height of 5 μm. The average predicted coefficient of error of the estimated total number of neurons was as followed: former vehicle: 0.056; former CBD: 0.056; current vehicle: 0.051; current CBD: 0.054.

## Discussion

CBD has been discussed as a therapy for neurodegenerative diseases including multiple sclerosis as well as diseases known for involving activation of the immune system and associated with oxidative stress (Iuvone et al., 2009; Krishnan et al., 2009; Scuderi et al., 2009; Booz, 2011). While most studies concentrate on THC, only a few studies investigate CBD treatment in healthy mice. However, there is evidence for many positive effects of prolonged CBD treatment on several brain-associated diseases like brain ischemia and epilepsy. Interestingly, positive effects of CBD treatment could be shown in mice with multiple sclerosis. CBD-treated mice with a dose of 5 mg/kg for 7–10 days reduced infiltration of leukocytes and the expression of cytokines (Mecha et al., 2013). Similarly, Patra et al. (2019) demonstrated a reduction of seizure burden as well as seizure severity in epilepsy models of rats and mice treated with CBD. In contrast to the probably most known constituent of the cannabis plant, THC, the mechanism of action for CBD remains still unclear, mainly because it involves several pharmacological targets (De Petrocellis and Di Marzo, 2010). Regarding the rising amount of CBD-containing lifestyle products (e.g., cosmetics, energy bars, drinks) more and more people consume CBD in their everyday life. This emphasizes the importance of studies in healthy rodents and humans.

Our study examines the influence of prolonged CBD treatment on healthy adult C57BL/6J mice and its consequences on behavior and hippocampal neuron numbers. Mice were divided into two treatment groups: a former treatment group and a current treatment group. The former treatment group received daily intraperitoneal injections of 20 mg/kg CBD-solution or vehicle-solution starting at the age of 3 months, while the current treatment group got the same treatment but started at the age of 5 months. Both groups started behavior testing at 6 months age. The aim of our study was to compare the influence of prolonged CBD treatment (current group) to the effects of prolonged CBD treatment with a washout period (former group) in adult mice.

It is described that there are sex differences in the modulation and expression of the endocannabinoid system. Cannabinoid exposure during adolescence has been shown to have long term consequences on brain and behavior (Andersen, 2003). Using cannabinoids during adolescence is more concerning because the endocannabinoid system is developed especially in this lifetime period and thus more vulnerable to exogenous insults as exposure of cannabinoids (Rice and Barone, 2000). It is known that gonadal hormones modulate the effects in cannabinoids in adult rodents, which were used in our study (Marusich et al., 2015). Several studies suggested that female rodents are more sensitive to the effects of THC than males (Craft et al., 2012; Marusich et al., 2014). Nevertheless, there is no evidence about the effects of CBD on the modulation of the endocannabinoid system. In our study, there was no gender difference regarding behavior (data not shown).

To evaluate the effect of chronic CBD treatment on reference and spatial memory, mice performed the MWM. No effect on spatial memory and long term memory could be observed. These findings correlate with the results of Fadda et al. (2004) who found no spatial learning impairments in CBD-treated rats in the MWM. Even at doses of up to 50 mg/kg CBD treatment had no effect on spatial working memory in these rats. Interestingly, research in a pharmacological mouse model of Alzheimer’s disease revealed positive effects of 20 mg/kg CBD treatment in the MWM (Martín-Moreno et al., 2011).

Learning in the MWM is highly dependent on the hippocampus (D’Hooge and De Deyn, 2001), therefore, the effect of CBD treatment on the neuron number in the CA1-region of the hippocampus was analyzed. No significant difference in the number of neurons or the volume of the hippocampus was detected after CBD treatment. Interestingly, Schiavon et al. (2014) showed that CBD treatment reduced MWM deficits and hippocampal neurodegeneration in response to brain ischemia in mice in a dose-dependent way. Likewise, Mori et al. (2017) observed a protective effect of acute treatment with 10 mg/kg CBD 30 min before and 3, 24 and 48 h after operation induced brain ischemia on neurodegeneration in the hippocampus. In a recently performed MRI study with regular cannabis users there was, in accordance to our findings, no difference in total hippocampal volume observed after treatment with CBD, while left subicular complex volume significantly increased from baseline to post-treatment, indicating a restorative effect of CBD on the subicular and CA1 subfields in cannabis users (Beale et al., 2018).

Similar to our findings in the MWM, there is no significant difference for NORT, concerning non-spatial learning and memory in C57BL/6J mice after prolonged CBD treatment. Both vehicle- and CBD-treated groups of former treatment showed a clear preference to the novel object. Our results are in line with Fagherazzi et al. (2012) who showed that CBD (5 mg/kg or 10 mg/kg for 14 days) does not affect memory of male adult rats in the NORT, neither were general parameters of behavior such as exploratory activity, locomotion and anxiety affected. Interestingly, their study also provides evidence that CBD might rescue memory impairments associated with brain disorder. This is in line with the results of Pazos et al. (2012) after hypoxic-ischemic injury via electrocoagulation.

Although CBD-treated mice traveled less distance in the OF test, they still had a clear preference to the novel object which excludes the confounding factor of locomotion. This stands in contrast with the findings of Viudez-Martínez et al. (2018) that CBD does not alter motor behavior 12 h after its administration of 30 mg/kg for 6 days in the OF test.

While CDB-treated mice showed an altered locomotion their motor performance in the Rotarod test was intact. Interestingly, Navarrete et al. (2018) observed a normalization of cannabis motoric withdrawal behavior signs in adult male C57BL/6J mice. This effect is similar to the observation in healthy volunteers where CBD blocked the anxiety produced by THC (Zuardi et al., 1982).

Considering the significance of the endocannabinoid system for energy metabolism and feeding behavior, we examined the effects of prolonged CBD administration on body weight gain in C57BL/6J mice. In the current study, long term CBD administration did not affect the food uptake and appetite of mice as CBD-treated mice displayed similar weight compared to same-aged vehicle-treated mice. These results are in contrast with previous findings from Riedel and colleges who reported that acute CBD treatment (10 mg/kg, one injection) induced a small although non-significant reduction in food intake and weight gain (Riedel et al., 2009). Furthermore, CBD treatment was shown to decrease the weight gain in rats (5 mg/kg for 14 days; Ignatowska-Jankowska et al., 2011). In adult male rats, oral administration of CBD (4.4 mg/kg) induced a significant reduction in total food intake over 4 h of test time (Farrimond et al., 2012). As we treated our mice for 42 days, these different findings may be the result of a habituation of the mice to the longer treatment time with CBD. To our knowledge, there is no data about the influence of CBD for human weight and stimulation of appetite, which makes further studies much more necessary. In contrast, there is evidence that synthetic THC (Dronabinol) is associated with an increase in weight when compared to placebo in HIV positive humans (Whiting et al., 2015).

A significance towards less distance traveled by CBD-treated mice in former and current treatment group could be observed in the OF, whereas the number of line crossings as a comparable parameter for distance in the DLB differed between current and former treatment. The OF test is a common measure of exploratory behavior and general activity with less focus on anxiety than the EPM. Increased anxiety probably results in less locomotion and in the OF test in the preference to stay in the periphery of the box (Ennaceur, 2014). Regarding the results of both tests, an increased anxiety behavior could be discussed as it is significantly shown in the current CBD-treated group compared to their vehicle-treated littermates. A moderate anxiolytic-like effect in the OF test in C57BL/6J mice at 50 mg/kg dose of CBD daily treated for 3 weeks, was observed by Long et al. (2010), whereas even a dose of 1 mg/kg significantly increased the time spent in the light compartment. After observing effects in chronic CBD treatment, Long et al. (2010) investigated the acute behavioral effects of CBD (1 mg/kg; 50 mg/kg) but no significant anxiolytic effects in the EPM could be found. In contrast to those findings, the current CBD-treated group in our study showed a significant decrease in the time spent in the open arms. Therefore, there is an anxiogenic-like effect of the current group in the EPM. Rats treated with lower doses of CBD (2.5, 5, 10 mg/kg) showed a significant increase in the entry ratio (open/total numbers of entries) which can be seen as an anxiolytic effect (Guimarães et al., 1990). Whereas a dose of 20 mg/kg had no effect compared to vehicle-treated male Wistar rats. This finding indicates that there is a limited range of anxiolytic doses and that the application and the following results are different in mice and rats. While CBD can be beneficial for treating anxiety, the beginning and the duration of the treatment is crucial and has to be considered and discussed in every treatment approach.

CBD treatment did not alter ASR in WT mice. Sensory gating describes the inhibition of a stimulus-related neuronal response if the stimulus is preceded by a subthreshold warning stimulus (Ally et al., 2006). Sensory gating can be measured using PPI, a method that can be studied with similar procedures in humans and rodents and reflects the ability to exclude sensory information from processing (Braff and Geyer, 1990). Current CBD altered sensorimotor gating, whereas former treated mice did not affect PPI. Our findings are well in line with the findings of Long et al. (2010) that there was no effect of CBD in ASR independent from the different acute treatment doses (1, 5, 10 or 50 mg/kg), although acute and chronic (1 mg/kg) CBD treatment altered PPI. In Swiss mice, CBD treatment with 5 mg/kg had no effect on PPI but dose-dependently increased the startle response and reversed a MK-801-induced PPI deficit (Long et al., 2006).

Interestingly, altered sensory gating has been well documented in different psychiatric conditions including schizophrenia (Gjini et al., 2011; Rohleder et al., 2016). However, the influences of CBD on ASR and PPI in animal models of schizophrenia have been inconsistent. CBD treatment reversed PPI disruptive effects of MK-801 and amphetamine in mice, while CBD had no effect in rats treated with MK-801 (Gururajan et al., 2011; Gomes et al., 2014; Pedrazzi et al., 2015).

The effects of CBD on startle response and PPI seem to be highly species-, strain- and dose-dependent. However, the mechanism involved in CBD action on PPI is not yet understood, highlighting the need for more studies to clarify the relationship between CBD and PPI.

To summarize our findings, no side effects in C57BL/6J mice were evident regarding memory, motoric abilities or anxiety behavior after long term CBD treatment. Based on these results it can be speculated that prolonged CBD treatment could be beneficial and safe for the treatment of a variety of conditions.

## Data Availability

The datasets generated for this study are available on request to the corresponding author.

## Ethics Statement

All animals were handled according to the guidelines of the Federation of European Laboratory Animal Science Association (FELASA) and approved by the “Lower Saxony State Office for Consumer Protection and Food Safety” (LAVES).

## Author Contributions

ES and FO performed experiments, analyzed data and wrote the manuscript. MS, JW, BS, MM and ML performed experiments. YB conceived and designed the project, performed experiments, analyzed data and wrote the manuscript. All authors contributed to revising the manuscript and approved the final version.

## Conflict of Interest Statement

The authors declare that the research was conducted in the absence of any commercial or financial relationships that could be construed as a potential conflict of interest.
